# Validation of the absolute renal risk of dialysis/death in adults with IgA nephropathy secondary to Henoch-Schönlein purpura: a monocentric cohort study

**DOI:** 10.1186/1471-2369-14-169

**Published:** 2013-08-01

**Authors:** Hesham Mohey, Blandine Laurent, Christophe Mariat, Francois Berthoux

**Affiliations:** 1 University Hospital of Saint-Etienne; Nephrology, Dialysis and Renal Transplantation Department, North Hospital, Saint-Etienne, France; 2 CHU de Saint-Etienne, Hôpital Nord; Service de Néphrologie, Dialyse et Transplantation Rénale, 42055 Saint-Etienne Cedex 2, France

**Keywords:** Immunoglobulin A, IgA nephropathy, Risk factors, Prediction of prognosis, Systemic glomerulonephritis, Henoch-Schönlein purpura nephritis

## Abstract

**Background:**

We established earlier the absolute renal risk (ARR) of dialysis/death (D/D) in primary IgA nephropathy (IgAN) which permitted accurate prospective prediction of final prognosis. This ARR was based on the potential presence at initial diagnosis of three major, independent, and equipotent risk factors such as hypertension, quantitative proteinuria ≥ 1 g per day, and severe pathological lesions appreciated by our local classification scoring ≥ 8 (range 0–20). We studied the validity of this ARR concept in secondary IgAN to predict future outcome and focused on Henoch-Schönlein purpura (HSP) nephritis.

**Methods:**

Our cohort of adults IgAN concerned 1064 patients with 101 secondary IgAN and was focused on 74 HSP (59 men) with a mean age of 38.6 at initial diagnosis and a mean follow-up of 11.8 years. Three major risk factors: hypertension, proteinuria ≥1 g/d, and severe pathological lesions appreciated by our global optical score ≥8 (GOS integrated all elementary histological lesions), were studied at biopsy-proven diagnosis and their presence defined the ARR scoring: 0 for none present, 3 for all present, 1 or 2 for the presence of any 1 or 2 risk factors. The primary end-point was composite with occurrence of dialysis or death before (D/D). We used classical statistics and both time-dependent Cox regression and Kaplan-Meier survival curve methods.

**Results:**

The cumulative rate of D/D at 10 and 20 years post-onset was respectively 0 and 14% for ARR = 0 (23 patients); 10 and 23% for ARR = 1 (N = 19); 27 and 33% for ARR = 2 (N = 24); and 81 and 100% (before 20 y) in the 8 patients with ARR = 3 (P = 0.0007). Prediction at time of diagnosis (time zero) of 10y cumulative rate of D/D event was 0% for ARR = 0, 10% for ARR = 1, 33% for ARR = 2, and 100% by 8.5y for ARR = 3 (P = 0.0003) in this adequately treated cohort.

**Conclusion:**

This study clearly validates the Absolute Renal Risk of Dialysis/Death concept in a new cohort of HSP-IgAN with utility to individual management and in future clinical trials.

## Background

In IgA nephropathy (IgAN), one difficulty is to predict accurately at time of diagnosis (by renal biopsy) the ultimate prognosis one or two decades later of the individual patient with the probability risk of reaching end-stage renal failure with the need for dialysis or to die before this event.

In primary IgAN, many risk factors predictive of progression have been described [1,2] and in a previous work [3], we focused on the following three consensual and major risk factors: occurrence of arterial hypertension (HT), amount of daily proteinuria, and severe renal lesions on optical microscopy (appreciated by local pathological scoring [4,5] or by the new Oxford classification [6]). These risk factors were simplified and dichotomized, before integration in an Absolute Renal Risk (ARR) score, which proved to be an overall accurate predictor of ultimate prognosis. These simplified risk factors, present or not at time of diagnosis, were:-HT (Yes or No); daily proteinuria ≥ 1 g/day (Yes or No);-and a global optical score, GOS ≥ 8 (Yes or No). We have previously defined the ARR score as the number of these risk factors present at diagnosis with four possibilities: 0, 1, 2, and 3. This ARR permitted us in a prospective study [3], including 332 patients with primary IgAN, to predict the cumulative incidence rate at 20 years post clinical onset of the combined final event, dialysis or death: 4% for ARR = 0; 9% for ARR = 1; 18% for ARR = 2; and 64% for ARR = 3. These findings were also validated in a retrospective historical cohort including 250 patients [3].

IgA nephropathies, defined as at least 1+ mesangial IgA deposits by immunofluorescence, are clinically divided in two groups:-primary IgAN, also called Berger’s disease which represent 90% of the cases, and-secondary IgAN observed in different clinical conditions: Henoch-Schönlein Purpura, HSP, alcoholic liver cirrhosis, some cases of Systemic Lupus Erythematosus, SLE, and few other rare conditions.

The goal of this retrospective observational study was to review all our cases of secondary IgAN and to apply our ARR score for an additional validation in another group of patients, and we focused on IGAN 2ary to HSP, which represented the majority of the cases.

## Methods

### The patients

We have reviewed all our adult cases of secondary IgAN collected from 1975 to 2010 and included with the following criteria: to have a renal biopsy showing at least 1+ mesangial IgA deposits and a minimum of 6 glomeruli available for optical microscopy (HSP clinical nephritis without biopsy were excluded), and a clinical classification among the following conditions: HSP with clinical purpura; overt clinical/pathological cirrhosis; systemic lupus erythematosus SLE; ankylosing spondylarthritis, AS; and few others. Finally during this period, we have registered 963 cases of primary IgAN and 101 secondary IgAN (9.5%, 101 over 1064). The aetiology of the secondary cases were: HSP in 74 (73.3%); liver cirrhosis in 19, SLE in 3, AS in 2, Goujerot-Sjögren syndrome in 1, systemic vasculitis in 1, and superimposed on a diabetic glomerulosclerosis in 1.

In this study, we report only on the 74 cases of IgAN secondary to HSP: 59 men (80%) and 15 women with at diagnosis a mean age of 38.6 (SD = 19.7) years and a median age of 37.1 (extremes: 5.9 to 74.6) years. All patients gave informed consent for the anonymous use of their personal health data.

It should be noted that this cohort was adequately treated with some heterogeneity to target these risk factors. Overall, 46% (34/74) of the patients received a treatment (over 6 months) with ACE inhibitors or ARBs for HT and/or heavy proteinuria; 63% of all patients who developed HT and 58% of patients with proteinuria ≥ 1 g/d received this treatment; the % of patients treated by ARR category (0, 1, 2, 3) was respectively 22, 47, 67, and 50. For steroid treatment, overall 44 patients were treated (59%) and the % by ARR category (0, 1, 2, 3) was respectively 35, 58, 75, and 88; the indication was severe pathological lesions (77%, 23/30) and/or heavy proteinuria at any time (81%, 29/36). The number of patients receiving additional therapy was 6, 3, and 6 respectively for immunosuppressive agents (4 Azathioprine, 1 Chlorambucil, and 1 Mabthera), plasma exchanges (mean of 6 sessions), and tonsillectomy.

### The methods

This is a retrospective monocentric study concerning an observational cohort of patients. Our hospital institutional review board (Comite de Protection des Personnes-Sud-Est 1) gave specific approval for this study.

Each patient chart was thoroughly reviewed with collection of clinical, biological, and pathological data at onset, at diagnosis and at last follow-up.

The risk factors studied were:-hypertension defined as blood pressure over 140/90 mmHg at different occasions or already treated with antihypertensive agents including diuretics;-daily proteinuria with a cut-off value of ≥1 g/day; and-global optical score, GOS, integrating all elementary lesions (glomerular index: 0 to 6, vascular index: 0 to 5, tubular index: 0 to 4 and interstitial index: 0 to 5); the cut-off value of ≥8 was previously extracted from ROC curve with dialysis as the final event.

We focused mainly on these risk factors: HT present or not at onset, at diagnosis, and at last follow-up. Blood pressure was regularly recorded with the antihypertensive treatment details; this variable was used only as dichotomous: present or absent. Daily proteinuria at diagnosis and at last follow-up with classification according to K-DOQI: <0.30 g/d; 0.30 to 0.99 g/d; 1.00 to 2.99 g/d; and ≥3 g/d; this variable was used both as continuous or dichotomous: <1 g/d or ≥1 g/d. The diagnostic renal biopsy was scored according to our local classification already described [4,5] with calculation of GOS value (scale from 0 to 20); this covariate was used both as continuous and dichotomous: <8 or ≥8 units. It is important to mention that 52 patients had no crescents (<5%), 14 with a low % of crescents (5 to 24%), and 8 with a high % of crescents (≥25%). Only one patient fulfilled the definition of rapidly progressive glomerulonephritis with more than 50% of crescents.

Time zero corresponded to disease onset with first renal signs: usually gross haematuria, microscopic haematuria, and/or proteinuria. For the calculation at time of diagnosis of the future risk of D/D 10 years later, time zero was set at time of the diagnostic biopsy.

The final combined primary event was the need for dialysis with an eGFR < 15 ml/mn/1.73 m^2^ S (stage 5D) or death occurring before. Reduction in GFR is a continuum from stage 1 to stage 5 with a cut-off value of <60 ml/mn/1.73 m^2^ which defined the start of chronic renal failure (CRF or CKD-stage 3 and up) and was used as a secondary event.

The absolute renal risk (ARR) at diagnosis was simply calculated by the number of risk factors present in each individual patient with 4 possibilities: 0 for none of these risk factors present, 3 for all these 3 risk factors present, and 1 or 2 for 1 or 2 any risk factors present among the 3 preselected.

### The statistics

We have used basic statistics with number and % for qualitative variables and mean (+/− SD) for continuous variables. Comparisons were done accordingly with contingency tables or unpaired T or U tests.

We used Cox regression models for survival without the event (dialysis/death) with both univariate or multivariate analyses (with different covariates: categorical or dichotomous or continuous). The survival curves were built according to the Kaplan-Meier method which accepts only categorical variables, and the curves were compared by the Logrank test. We have also done these statistics with dialysis alone as end-point. All tests were done with Statview 5 or SPSS 19 softwares.

## Results

### Baseline data at diagnosis

All 74 patients presented with cutaneous purpura at least on one occasion with the classical characteristics; the initial presentation was associated with arthralgia in 57 (77.0%) together with abdominal pain in 33 (44.6%) leading to the diagnosis of Henoch-Schönlein purpura. Renal involvement occurred at the same time of purpura or within few weeks: microscopic haematuria was detected at time of biopsy in all patients but one, and gross haematuria was symptomatic in 29 (39.2%); significant proteinuria ≥0.30 g/d was detected in 54 patients (73.0%).

The risks factors already present at time of biopsy (diagnosis) are detailed in Table 1: 32 with proteinuria ≥1 g/d (43.2%); 29 with hypertension (39.2%); and 30 with severe renal lesions with GOS ≥8 (40.5%). Progression of the disease was demonstrated by both the number of patients developing HT during the disease course: 22 at disease onset (29.7%); 29 at time of diagnosis (39.2%), and 49 at last FU or at final event (66.2%), and the number of patients with CRF (CKD-3+) already at diagnosis 23% (17/74) and at last follow-up 36.5% (27/74), and the number of patients reaching the primary end-point (D/D): 2.7% at diagnosis (2/74), and 25.7% at last FU (19/74 with 15 on dialysis and 4 dying before dialysis). The mean overall exposure time to the final risk was 11.8 (SD = 10.6) years with 75% followed more than16.2 years.

**Table 1:** Characteristics of the HSP-IgAN patients at diagnosis and at last follow-up

**Items**	**Units**	**At diagnosis**	**At last follow-up**	**Test; P value**
**Age:** mean (SD)	years	38.6 (19.7)	46.8 (18.7)	
Time interval: m(SD)	years	3.6 (8.1)	8.2 (7.2)	
**Gross Haematuria:**	N (%)	29 (39.2%)	29 (39.2%)	
**Proteinuria:**				
Mean (SD):	g/d	1.29 (1.56)	0.43 (0.68)	T = −4.8; <0.0001
Median (extremes):	g/d	0.80 (0.00-10.00)	0.18 (0.00-3.66)	
Class (g/d):				
<0.30 or absent	N (%)	20 (27.0%)	44 (59.5%)	
0.30–0.99	N (%)	22 (29.7%)	21 (28.4%)	X^2^ = 15.0; NS (0.09)
1.00–2.99	N (%)	19 (25.7%)	7 (9.5%)	
≥ 3.00	N (%)	13 (17.6%)	2 (2.7%)	
**Proteinuria ≥1 g/d**	N (%)	**32 (43.2%)**	9 (12.2%)	
**Hypertension:** yes	N (%)	**29 (39.2%)**	49 (66.2%)	X^2^ = 24.3; <0.0001
SBP in HT + ve: m (SD)	mm Hg	140.2 (16.9)	139.5 (17.2)	
SBP in HT-ve: m (SD)	mm Hg	128.4 (19.0)	120.2 (15.7)	
DBP in HT + ve: m (SD)	mm Hg	84.8 (13.5)	82.9 (9.2)	
DBP in HT-ve: m (SD)	mm Hg	75.8 (12.5)	76.6 (10.0)	
**Pathology/GOS (0–20):**				
Mean (SD)	units	7.17 (2.75)	/	
Median (range)	units	7.00 (2–16)	/	
**GOS ≥ 8**	N (%)	**30 (40.5%)**	/	
GVTI indices: m (SD)	G = 3.38 (1.40)	V = 1.95 (1.06)	T = 0.87 (0.68)	I = 0.97 (0.74)
**End-point/eGFR:**				
Mean (SD)	ml/mn/1.73 m^2^ S	82.2 (34.0)	60.0 (35.2)	T = 6.3;<0.0001
Median (extremes)	ml/mn/1.73 m^2^ S	85.3 (7.1-200.4)	68.7 (5.8-147.0)	
eGFR staging:				
Stage 1: ≥ 90	N (%)	30 (40.5%)	14 (18.9%)	
Stage 2: 60–89	N (%)	27 (36.5%)	33 (44.6%)	
Stage 3: 30–59	N (%)	11 (14.9%)	9 (12.2%)	X^2^ = 60.3; <0.0001
Stage 4: 15–29	N (%)	3 (4.1%)	2 (2.7%)	
Stage 5: <15	N (%)	3 (4.1%)	16 (21.6%)	
CKD-3+ (eGFR < 60)	N (%)	17 (23.0%)	27 (36.5%)	X^2^ = 25.5; <0.0001
**Dialysis**	N (%)	**2 (2.7%)**	**15 (20.3%)**	
Death	N (%)	0 (0.0%)	4 (5.4%)	
**Dialysis/Death**	N (%)	**2 (2.7%)**	**19 (25.7%)**	X^2^ = 6.0; =0.01

Most important parameters are bolded.

The distribution of ARR score is given in Table 2 and showed a significant difference according to age at diagnosis: more patients with score [0 + 1] in younger patients (<25y) contrasting to more patients with score [2 + 3] in older patients (>50y).

**Table 2:** Absolute renal risk score distribution at diagnosis in HSP-IgAN patients

**Nb of risk factors**	**ARR score (risk level)**	**Overall distribution**	**Distribution in men**	**Distribution in women**	**Age at diagnosis:**			**D/D event N (%)**
					**(<25)**	**(25–50)**	**(>50)**	
0	**0 (very low)**	23 (31.1%)	18 (30.5%)	5 (33.3%)	15	6	2	1 (4.3%)
1	**1 (low)**	19 (25.7%)	13 (22.0%)	6 (40.0%)	2	9	8	3 (15.8%)
2	**2 (high)**	24 (32.4%)	20 (33.9%)	4 (29.3%)	4	8	12	9 (37.5%)
3	**3 (very high)**	8 (10.8%)	8 (13.6%)	0 (0.0%)	3	3	2	6 (75.0%)
			X^2^ = 3.76	P = NS	X^2^ = 20.7; P = 0.002			

The % of patients with HT according to ARR 0, 1, 2, or 3, was at diagnosis 0, 37, 58 and 100 contrasting at last follow-up with 30, 63, 92, and 100 respectively The % of patients with proteinuria ≥1 g/d was at diagnosis 0, 37, 71, and 100 and at any time 4, 47, 75, and 100 respectively for ARR 0, 1, 2, and 3. The % of patients with GOS ≥8 at diagnosis was respectively 0, 26, 71, and 100 for ARR 0, 1, 2, and 3.

### Association of ARR score at diagnosis with progression to final event

Overall, 15 patients reached ESRF and needed dialysis and 4 patients died before dialysis. These deaths concerned 3 men and 1 woman; causes were cardiovascular in 2, infection in 1 and other in 1; these patients died at age 49, 66, 77 and 85 years.

The overall cumulative incidence rate of dialysis/death event (Table 3) was respectively at 0, 5, 10, 15, and 20 years post-onset, 0% (74 at risk), 9% (50 at risk), 19% (37 at risk), 25% (20 at risk), and 29% (15 at risk). By Cox regression analysis, the ARR score at diagnosis predicted nicely survival without D/D (X^2^ = 12.1; P = 0.007): the relative risk with 95% confidence interval was RR =0.04 (0.005-0.35) for ARR = 0; RR = 0.13 (0.03-0.68) for ARR = 1; and RR = 0.29 (0.09-0.96) for ARR = 2 as compared to RR = 1 for ARR = 3. Similar data was obtained for prediction of dialysis alone: X^2^ = 13.4; P = 0.004; RR = 0.04 (0.01-0.34) for ARR = 0, RR = 0.07 (0.01-0.61) for ARR = 1, RR = 0.28 (0.09-0.88) for ARR = 2 as compared to 1 for ARR = 3 (see Additional file 1: Table S1).

**Table 3:** Cumulative incidence rate of dialysis/death (D/D) in HSP-IgAN patients

**D/D Cumulative incidence rate**	**Time = 0 %**	**Time = 5 y %**	**Time = 10 y %**	**Time =15 y %**	**Time = +20 y %**
	**(at risk)**	**(at risk)**	**(at risk)**	**(at risk)**	**(at risk)**
**After Disease Onset:**					
Overall	0% (74)	9% (50)	**19%** (37)	25% (20)	29% (15)
ARR = 0	0% (23)	0% (18)	**0%** (16)	0 % (9)	14% (6)
ARR = 1	0% (19)	0% (13)	**10%** (8)	23% (2)	/
ARR = 2	0% (24)	15% (15)	**27%** (12)	33% (8)	33% (6)
ARR = 3	0% (8)	42% (4)	**81%** (1) at 8.5y	/	/
**After Diagnosis:**					
Overall	0% (74)	13% (42)	**25%** (30)	40% (9)	/
ARR = 0	0% (23)	0% (15)	**0%** (12)	0% (5)	/
ARR = 1	0% (19)	0% (11)	**10%** (8)	25% (2)	/
ARR = 2	0% (24)	14% (14)	**33%** (10)	61% (2)	/
ARR = 3	0% (8)	71% (2)	**100%** (2) at 8.5y	/	/

Most important parameters are bolded.

**Figure 1:**
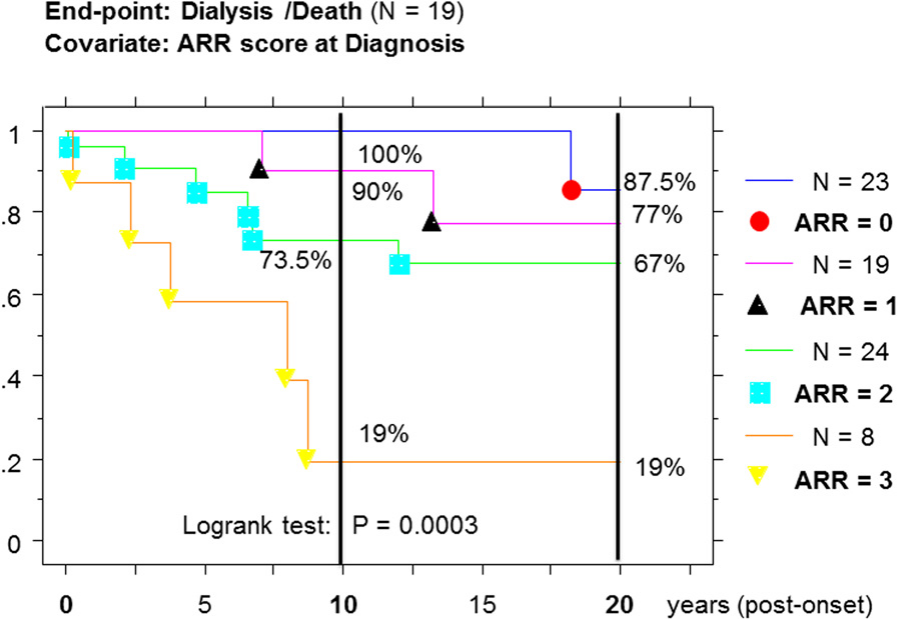
Kaplan-Meier survival curves without Dialysis/Death event and according to Absolute Renal Risk score at Diagnosis in HSP-IgAN patients (time zero is disease onset).

By Kaplan-Meier method, the survival curves without D/D (Figure 1) were nicely stratified from ARR = 0 (best survival) to ARR = 3 (worse survival) and significantly different by the Logrank test (X^2^ = 18.5; P = 0.0003). The cumulative incidence rate of D/D event at 10 and 20 y post-onset, were respectively 0% and 14% for ARR = 0; 10% and 23% for ARR = 1; 27% and 33% for ARR = 2; and finally 81% at 10y for ARR = 3.

### Capacity of predicting ultimate progression by ARR scoring at diagnosis

To achieve this goal, we set up time zero at time of diagnosis (renal biopsy) and recalculate both Cox regression and Kaplan-Meier survival.

By Cox analysis, the ARR score predicted progression to D/D (X^2^ = 21.0; P = 0.0001); the respective RR was 0.02 (CI: 0.002-0.14; P = 0.002) for ARR = 0, 0.06 (CI: 0.01-0.28; P = 0.0003) for ARR = 1, and 0.17 (CI: 0.05-0.53; P = 0.003) for ARR = 2, and all as compared to 1 for ARR = 3.

**Figure 2:**
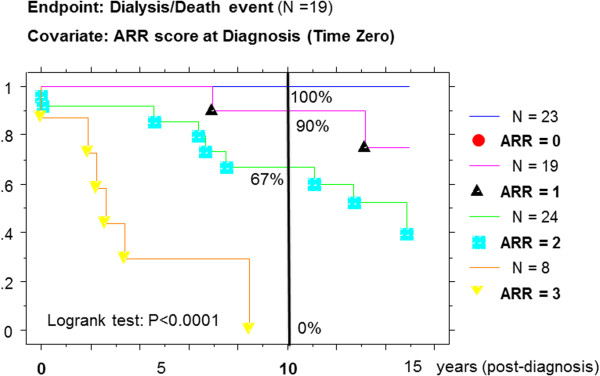
Prospective Kaplan-Meier survival curves without Dialysis/Death (D/D) event and according to Absolute Renal Risk score at Diagnosis in HSP-IgAN patients (time zero is biopsy-proven diagnosis).

By Kaplan-Meier method, the survival curves without D/D (Figure 2) were also nicely stratified and significantly different by the Logrank test (X^2^ = 37.6; P < 0.0001). The cumulative incidence rate of D/D event at 10 years post-diagnosis was respectively 0% for ARR = 0 (12 at risk), 10% for ARR = 1 (8 at risk), 33% for ARR = 2 (10 at risk), and 100% reached D/D by 8.5 years for ARR = 3.

Similar data was obtained for prediction of Dialysis alone by Logrank test (X^2^ = 44.30; P < 0.0001) with cumulative incidence rate of dialysis at 10 y of 0% for ARR = 0, 10% for ARR = 1, 25% for ARR = 2, and 100% reached dialysis by 7 years.

## Discussion

The strict application of our ARR score to a moderate-size cohort of adult IgAN secondary to HSP demonstrated that the ARR score was able to predict at time of evaluation (diagnosis) the ultimate prognosis 10 and 20 years post-onset with a good stratification from ARR = 0 to ARR = 3. In this regard, our ARR scoring developed originally in a large prospective cohort of primary IgAN [3] (N = 332) was validated in this external cohort (but from the same group) after being validated in an internal retrospective cohort of 250 cases of primary IgAN. Recently a Norvegian group confirmed the validity of our Absolute Renal Risk in 633 primary IgAN patients and can serve as an external validation of our predictive model (Knoop T, Vikse BE, Svarstad E et al.; unpublished data).

The present study has some limitations:-the relative small size population should be balanced by the fact that HSP-nephritis represents at most 10% of all IgA nephropathies;-it is clearly a retrospective study but with prospective and homogenous collection of data in the patients chart similar to our prospective cohort of primary IgAN;-and this monocentric study warrants more homogeneity in the diagnosis and management of the patients than a multicentric study, which however will have permitted to include more patients.

The question of including a marker of renal function in our model was already discussed in our original paper [3]:-we thought that it was not appropriate to use the same item such as GFR both as risk factor and as primary end-point (dialysis with GFR in stage V);-nevertheless, eGFR staging (1 to 5) at diagnosis was tested but substituted to HT and proteinuria ≥1 g/d in the subgroup of patients with already chronic renal failure, CRF (eGFR < 60 ml/mn/1.73). In this small cohort, we found no significant impact of CRF tested as additional dichotomous covariate (yes or no) together with either ARR (0 to 3) or with the 3 covariates (HT, proteinuria ≥ 1 g/d, and GOS ≥ 8); see Additional file 1: Table S1.

It is remarkable to notice the similarities between this HSP cohort and our prospective cohort of primary IgA nephropathy, IGAN-STET-CO: -The distribution of the three major risk factors was very similar: respectively for HSP and IGAN-STET-CO, 39% versus 36% for HT present at diagnosis; 43% versus 30% for proteinuria ≥ 1 g/d at diagnosis; and 40% versus 36% for GOS ≥8 (the mean GOS values were also very closed: 7.17 versus 7.00); -The values of eGFR at diagnosis and at last FU were also very closed with similar staging distribution: 23% versus 26% of patients in CKD-3+; but finally at last follow-up, we have observed D/D events in 26% of HSP-IgAN patients versus 14% in primary IgAN patients; -In the Cox analyses," the respective weight of each risk factor was difficult to appreciate totally in this paper because of the limited number of patients in this HSP cohort; the β/SE ratios for each dichotomous factor was in the following order: greater for the absence of GOS ≥ 8, then absence of proteinuria ≥1 g/d and last absence of HT. From the difference in the number of final D/D events between HSP-IgAN and primary IgAN, we could not derived any conclusion because one cohort was retrospective and the other prospective; in addition, patients with HSP-IgAN received less and later therapy with ACEI or ARBs which could have decreased survival without D/D.

Many clinical studies concerning HSP nephritis in adults have been published [7-11]; few focused on long-term prognosis with characterization of risk factors [12-16]; and others on comparisons between adults and children [17-21]. The long-term prognosis of HSP nephritis was found worse in adults than in children/adolescent. The main clinical risk factors were also proteinuria over 1 g/d and hypertension. Just recently, an observational study [22] demonstrated that the final prognosis in adults was similar in HSP-IgAN and in primary IgAN, when fully matched by propensity score method (but HT was included as a comorbid condition in the matching and not as an individual risk factor for progression).

Concerning renal pathology, the most predictive optical glomerular lesions for end-stage renal failure were the presence of crescents and endocapillary hypercellularity. To our knowledge, there has been no specific use of Oxford classification in SHP-IgAN for potential validation, but a recent study in primary IgA nephropathy confirmed that the % of crescents was a poor prognosis marker and should be included in the revised Oxford classification [23] for primary IgAN. We have new information [24] (plus Berthoux F et al., unpublished data) in a subgroup of 151 patients with primary IgAN concerning the equivalence between our Local classification (GOS) and the International/Oxford classification (MEST):-the linear correlation is excellent (R = 0.79; P < 0.0001) with the formula “MEST = (+0.256*GOS)-0.725 “;-the respective range is 0 to 20 for GOS and 0 to 5 for MEST;-MEST ≥2 correspond to GOS ≥8;-and the respective power of MEST and GOS in predicting dialysis is similar in multivariate Cox and multivariate logistic regressions analyses with continuous or dichotomous covariates.

It should be mentioned that the % of crescents was integrated in the glomerular index of our local classification with final higher GOS values. By monovariate Cox regression for prediction of dialysis alone, the variable “% of crescents” had a significant effect (p > 0.0001).

The similarities between primary IgAN and HSP-IgAN have been stressed since a long time, but recently the common pathogenesis was demonstrated [25,26] with data on the autoantigen, galactose-deficient IgA1, and specific autoantibodies (IgG and IgA subclasses); a common genetic background for this galactose-deficient IgA1 was also described [27].

It is out of our scope to review the treatment of HSP-IgAN [28] but obviously the treatment should target the risk factors when present: adequate control of hypertension with blood pressure below 130/80; reduction of proteinuria with ACE inhibitors and ARB’s; steroid treatment for severe renal lesions eventually associated to immunosuppressive agents.

In our study, the use of ACEI and/or ARBs was limited: overall respectively 63% and 58% of all patients with HT at anytime (31/49) and proteinuria ≥1 g/d at anytime (21/36) received such treatment; however in the subgroup of patients diagnosed since 1990 (N = 49), this % rose to 74 and 68% respectively. Nevertheless, survival without dialysis/death was strictly similar in the two subgroups: <1990 versus ≥1990.

## Conclusion

In a retrospective cohort of 74 adult patients with IgA nephropathy secondary to Henoch-Schönlein Purpura, we have validated the Absolute Renal Risk concept in the prediction at time of diagnosis of future outcome (dialysis/death or dialysis alone).

## Abbreviations

ARR: Absolute renal risk; AS: Ankylosing spondylarthritis; CRF: Chronic renal failure; D/D: Dialysis or death; eGFR: Estimated glomerular filtration rate; HSP: Henoch-Schönlein purpura; HT: Arterial hypertension; IgAN: IgA nephropathy; ROC: Receiver-operator curve; SLE: Systemic lupus erythematosus.

## Competing interests

No conflict of interest for any of the authors and no financial disclosure.

## Authors’ contributions

HM and FB reviewed the charts of the patients, collected all data, and performed the different statistics. BL reviewed all biopsies of the patients with scoring according to our local classification. CM and FB designed the study, wrote, corrected and discussed the manuscript. All authors read and approved the final manuscript.

## Pre-publication history

The pre-publication history for this paper can be accessed here:

http://www.biomedcentral.com/1471-2369/14/169/prepub

## Supplementary Material

Additional file 1: Table S1 Cox regression analyses for prediction of Dialysis alone. Click here for file

## References

[B1] Donadio JV Grande JP IgA nephropathy N Engl J Med 2002 347 738 748 10.1056/NEJMra020109 12213946

[B2] Barratt J Feehally J IgA nephropathy J Am Soc Nephrol 2005 16 2088 2097 10.1681/ASN.2005020134 15930092

[B3] Berthoux F Mohey H Laurent B Mariat C Afiani A Thibaudin L Predicting the risk for dialysis or death in IgA nephropathy J Am Soc Nephrol 2011 22 752 761 10.1681/ASN.2010040355 21258035 PMC3065230

[B4] Alamartine E Sabatier JC Berthoux FC Comparison of pathological lesions on repeated renal biopsies in 73 patients with primary IgA glomerulonephritis: value of quantitative scoring and approach to final diagnosis Clin Nephrol 1990 34 45 51 2225552

[B5] Alamartine E Sabatier JC Guerin C Berliet JM Berthoux F Prognostic factors in mesangial IgA glomerulonephritis : an extensive study with univariate and multivariate analyses Am J Kidney Dis 1991 18 12 19 2063844 10.1016/s0272-6386(12)80284-8

[B6] Cattran DC Coppo R Cook HT Feehally J Roberts IS Troyanov S Alpers CE Amore A Barratt J Berthoux F Bonsib S Bruijn JA D’Agati V D’Amico G Emancipator S Emma F Ferrario F Fervenza FC Florquin S Fogo A Geddes CC Groene HJ Haas M Herzenberg AM Hill PA Hogg RJ Hsu SI Jeannette JC A working group of the International IgA Nephropathy Network, The Renal Pathology Society The Oxford classification of IgA nephropathy: rationale, clinicopathological correlations, and classification Kidney Int 2009 76 534 545 10.1038/ki.2009.243 19571791

[B7] Nakamoto Y Asano Y Dohi K Fujioka M Iida K Kibe Y Hattori N Takeuchi J Primary IgA glomerulonephritis and Schönlein-Henoch purpura nephritis: clinicopathological and immunohistological characteristics Q J Med 1978 47 495 516 375278

[B8] Roth DA Wilz DR Theil GB Schönlein-Henoch syndrome in adults Q J Med 1985 55 145 152 3889976

[B9] Lee HS Koh HI Kim MJ Rha HY Henoch-Schönlein nephritis in adults: a clinical and morphological study Clin Nephrol 1986 26 125 130 3769227

[B10] Szeto CC Choi PC To KF Hui J Chow KM Leung CB Lui SF Mac-Moune LF Grading of acute and chronic renal lesions in Henoch-Schönlein purpura Mod Pathol 2001 14 635 640 10.1038/modpathol.3880364 11454994

[B11] Kellerman PS Henoch-Schönlein purpura in adults Am J Kidney Dis 2006 48 1009 1016 10.1053/j.ajkd.2006.08.031 17162160

[B12] Fogazzi GB Pasquali S Moriggi M Casanova S Damilano I Mihatsch MJ Zucchelli P Ponticelli C Long-term outcome of Schönlein-Henoch nephritis in the adult Clin Nephrol 1989 31 60 66 2646052

[B13] Tancrede-Bohin E Ochonisky S Vignon-Pennamen MD Flageul B Morel P Rybojad M Schönlein-Henoch purpura in adult patients. Predictive factors for IgA glomerulonephritis in a retrospective study of 57 cases Arch Dermatol 1997 133 438 442 10.1001/archderm.1997.03890400034005 9126006

[B14] Pillebout E Thervet E Hill G Alberti C Vanhille P Nochy D Henoch-Schönlein purpura in adults: outcome and prognostic factors J Am Soc Nephrol 2002 13 1271 1278 10.1097/01.ASN.0000013883.99976.22 11961015

[B15] Rauta V Törnroth T Grönhagen-Riska C Henoch-Schönlein nephritis in adults: clinical features and outcome in Finnish patients Clin Nephrol 2002 58 1 8 12141401 10.5414/cnp58001

[B16] Shrestha S Sumingham N Tan J Alhous H Mc William L Ballardie F Henoch-Schönlein purpura with nephritis in adults: adverse prognostic indicators in a UK population Q J Med 2006 99 253 265 10.1093/qjmed/hcl034 16565522

[B17] Coppo R Mazzucco G Cagnoli L Lupo A Schena FP Long-term prognosis of Henoch-Schönlein nephrit in adults and children. Italian Group of Renal Immunopathology Collaborative Study on Henoch-Schönlein purpura Nephrol Dial Transplant 1997 12 2277 2283 10.1093/ndt/12.11.2277 9394311

[B18] Chang WL Yang YH Wang LC Lin YT Chiang BL Renal manifestations in Henoch-Schönlein purpura: a 10-year clinical study Pediatr Nephrol 2005 20 1269 1272 10.1007/s00467-005-1903-z 15947991

[B19] Coppo R Andrulli S Amore A Gianoglio B Conti G Peruzzi L Locatelli F Cagnoli L Predictors of outcome in Henoch-Schönlein nephritis in children and adults Am J Kidney Dis 2006 47 993 1003 10.1053/j.ajkd.2006.02.178 16731294

[B20] Uppal SS Hussain MA Al-Raqum HA Nampoory MR Al-Saeid K Al-Assousi A Abraham M Malaviya AN Henoch-Schönlein’s purpura in adults versus children/adolescents: a comparative study Clin Exp Rheumato 2006 24 S26 S30 16859592

[B21] Hung SP Yang YH Lin YT Wang LC Lee JH Chiang BL Clinical manifestations and outcomes of Henoch-Schönlein purpura: comparison between adults and children Pediatr Neonatol 2009 50 162 168 10.1016/S1875-9572(09)60056-5 19750891

[B22] Oh HJ Ahn SV Yoo DE Kim SJ Shin DH Lee MJ Kim HR Park JT Yoo TH Kang SW Choi KH Han SH Clinical outcomes, when matched at presentation, do not vary between adult-onset Henoch-Schönlein purpura nephritis and IgA nephropathy Kidney Int 2012 82 1304 1312 10.1038/ki.2012.302 22895518

[B23] Katafuchi R Ninomiya T Nagata M Mitsuiki K Hirakata H Validation study of Oxford classification of IgA nephropathy: the significance of extracapillary proliferation Clin J Am Soc Nephrol 2011 6 2806 2813 10.2215/CJN.02890311 22157710 PMC3255377

[B24] Alamartine E Sauron C Laurent B Sury A Seffert A Mariat C The use of Oxford classification of IgA nephropathy to predict renal survival Clin J Am Soc Nephrol 2011 6 2384 2388 10.2215/CJN.01170211 21885791 PMC3359557

[B25] Lau KK Wyatt RJ Moldoveanu Z Tomana M Julian BA Hogg RJ Lee JY Huang WQ Mestecky J Novak J Serum levels of galactose-deficient IgA in children with IgA nephropathy and Henoch-Schönlein purpura Pediatr Nephrol 2007 22 2067 2072 10.1007/s00467-007-0623-y 17943324

[B26] Lau KK Suzuki H Novak J Wyatt RJ Pathogenesis of Henoch-Schönlein purpura nephritis Pediatr Nephrol 2010 25 19 26 10.1007/s00467-009-1230-x 19526254 PMC2778786

[B27] Kiriluk K Moldoveanu Z Sanders JT Eison TM Suzuki H Julian BA Novak J Gharavi AG Wyatt RJ Aberrant glycosylation of IgA1 is inherited in both pediatric IgA nephropathy and Henoch-Schönlein purpura nephritis Kidney Int 2011 80 79 87 10.1038/ki.2011.16 21326171 PMC3641561

[B28] Davin JC Henoch-Schönlein purpura nephritis: pathophysiology, treatment, and future strategy Clin J Am Soc Nephrol 2011 6 679 689 10.2215/CJN.06710810 21393485

