# Supplementary material for: Validation of the absolute renal risk of dialysis/death in adults with IgA nephropathy secondary to Henoch-Schönlein purpura: a monocentric cohort study
Source: BMC Nephrol. 2013 Aug 1;14:169. doi: 10.1186/1471-2369-14-169 (PMC3733957; doi:10.1186/1471-2369-14-169)
Supplement: Supplementary file 1 [file 1471-2369-14-169-S1.doc]

ITEM	Status	β/SE	X^2	P	RR	95% CI
**Cox**	**Mono**	**Variate**	**Analysis :**			
**GOS≥8**	Absence	-2.77	7.68	**0.006**	**0.12**	0.03-0.54
**Prot≥1g/d**	Absence	-2.27	5.16	**0.02**	**0.26**	0.08-0.83
**HT**	Absence	-2.16	4.37	**0.03**	**0.29**	0.10-0.89
**ARR**<br>**ARR= 0**<br>**ARR= 1**<br>**ARR= 2**	Overall	/<br>-2.95<br>-2.42<br>-2.17	13.37<br>8.68<br>5.85<br>4.72	**0.004**<br>**0.003**<br>**0.02**<br>**0.03**	**/**<br>**0.04**<br>**0.07**<br>**0.28**	/<br>0.01-0.34<br>0.01-0.61<br>0.09-0.88
**CRF**	Absence	-1.82	3.31	**0.07**	**0.36**	0.12-1.08
**Cox**	**Multi**	**Variate**	**Analysis1:**			
**GOS≥8**	Absence	-2.37	5.62	**0.02**	**0.13**	0.02-0.70
**Prot≥1g/d**	Absence	-1.82	3.31	**0.07**	**0.32**	0.10-1.10
**HT**	Absence	-2.03	4.12	**0.04**	**0.21**	0.05-0.95
**CRF**	Absence	+1.28	1.64	**NS**	**2.78**	0.58-13.27
**Cox**	**Multi**	**Variate**	**Analysis 2:**			
**ARR**<br>**ARR= 0**<br>**ARR= 1**<br>**ARR= 2**	Overall	/<br>-3.15<br>-2.71<br>-2.33	12.21<br>9.89<br>7.33<br>5.42	**0.007**<br>**0.002**<br>**0.007**<br>**0.02**	/<br>**0.02**<br>**0.03**<br>**0.15**	/<br>0.001-0.21<br>0.002-0.38<br>0.03-0.74
**CRF**	Absence	+1.25	1.56	**NS**	**2.80**	0.56-14.15

Supplementary Table 1: Cox regression analysis (Monovariate and Multivariate) for prediction of dialysis alone (15 events) using 5 different covariates: GOS≥8, Proteinuria≥1g/d, Hypertension (HT), Absolute Renal Risk score, ARR (0, 1, 2, 3) and Chronic Renal Failure (CRF; eGFR<60ml/mn/1.73m^2).
